# Bio-Organic Fertilizers and Microbial Biostimulants in Maize–Cereal Cropping Systems

**DOI:** 10.3390/plants15142120

**Published:** 2026-07-09

**Authors:** Gurwinder Singh, Baljinder Singh, Munish Sharma, Ankit Saini, Mehmet Ramazan Rişvanlı, Gökhan Boyno, Younes Rezaee Danesh, Rosa Porcel, José M. Mulet

**Affiliations:** 1 Department of Agronomy, Faculty of Agriculture, Guru Kashi University, Talwandi Sabo, Bathinda 151302, India; 2 School of Agricultural Sciences, Baddi University of Emerging Sciences and Technology, Solan 173205, India; 3 Department of Agronomy, Dr Khem Singh Gill Akal College of Agriculture, Eternal University, Baru Sahib 173101, India; 4 Department of Plant Protection, Faculty of Agriculture, Van Yüzüncü Yıl University, 65090 Van, Türkiye; 5 Instituto de Biología Molecular y Celular de Plantas, Universitat Politècnica de València-Consejo Superior de Investigaciones Científicas, 46022 Valencia, Spain

**Keywords:** bio-organic fertilizers, microbial biostimulants, maize-wheat rotation, crop diversification, soil health, nutrient-use efficiency, abiotic stress, biotic stress

## Abstract

Sustaining maize–cereal production under intensive agricultural systems is becoming increasingly challenging because of declining soil quality, low nutrient-use efficiency, and escalating abiotic and biotic stresses. In many regions, long-term rice–wheat cultivation has intensified these constraints, prompting greater interest in diversified production systems such as maize–wheat rotations. Within this context, bio-organic fertilizers (BOF) and microbial biostimulants are increasingly recognized not merely as nutrient inputs but as biologically active regulators of plant–soil interactions. This review synthesizes current knowledge on the role of BOF in maize–cereal production systems, with particular emphasis on maize–wheat rotations. It examines their effects on crop productivity, soil health, nutrient dynamics, and resilience to abiotic and biotic stresses. Current evidence indicates that BOF and microbial biostimulants improve crop performance through interconnected mechanisms, including nutrient mobilization, root development, rhizosphere regulation, and microbial-mediated soil processes. Collectively, these mechanisms enhance nutrient-use efficiency, strengthen stress tolerance, and contribute to more stable crop yields. Importantly, the benefits of BOF extend beyond single growing seasons by promoting soil biological activity, nutrient availability, and system resilience across successive crop cycles. Overall, BOF should be regarded not simply as alternatives to mineral fertilizers but as multifunctional regulators of plant–soil processes that support the sustainability of maize–wheat and other cereal-based cropping systems.

## 1. Introduction

Maize (*Zea mays* L.) and wheat (*Triticum aestivum* L.) are two of the most widely cultivated cereal crops in the world and play a central role in global food security [1,2]. In many cereal-growing regions, long-term reliance on mineral fertilizers has increased yields, but often at the cost of declining soil biological quality, lower nutrient-use efficiency, and greater environmental losses [3,4,5]. At the same time, drought, salinity, heat stress, nutrient imbalance, pathogens, and herbivores continue to exert considerable pressure on crop productivity, particularly in intensive agricultural systems where ecological buffering capacity has been diminished [6,7,8,9]. This problem is particularly evident in cereal-based production systems. Repeated rice–wheat cultivation, for example, has been associated with soil fertility decline, inefficient resource use, and increasing environmental pressure [10,11]. Consequently, crop diversification has attracted increasing attention as a sustainable strategy to improve resource-use efficiency while maintaining agricultural productivity [12]. Among the available alternatives, maize-wheat rotation has emerged as one of the most promising systems because it can reduce pressure on natural resources while improving residue decomposition, nutrient cycling, and rhizosphere functioning compared with less diversified cereal systems [13,14,15,16]. Within this context, bio-organic fertilizers (BOF) and microbial biostimulants are increasingly recognized not only as nutrient inputs but also as biologically active regulators of plant–soil interactions. These biological inputs include plant growth-promoting rhizobacteria (PGPR), plant growth-promoting fungi (PGPF) such as *Trichoderma* spp., arbuscular mycorrhizal fungi (AMF), composts, humic substances, biochar, and other biologically derived amendments. Collectively, these materials influence plant performance through interconnected processes including nutrient mobilization, root development, rhizosphere regulation, microbial interactions, stress mitigation, and, in some cases, pathogen suppression and defense priming [3,17,18,19,20,21,22,23]. Unlike many previous reviews that summarize the general effects of microbial inoculants across diverse crop species, the present review focuses specifically on maize–wheat and related maize–cereal production systems. These systems possess distinctive agronomic characteristics, including high nutrient demand across successive cereal crops, contrasting root architectures, substantial residue inputs, and strong dependence on maintaining soil biological functioning throughout crop rotations. Consequently, the establishment, persistence, and agronomic performance of BOF are influenced not only by microbial characteristics but also by the ecological continuity created within maize–wheat production systems. Synthesizing current knowledge within this specific cropping-system context therefore provides practical insights that extend beyond generalized evaluations of microbial biofertilizers in individual crops.

Although numerous studies have demonstrated the beneficial effects of these biological inputs, the available evidence remains fragmented. Most published studies focus on individual aspects, such as nutrient acquisition, stress tolerance, pathogen suppression, or soil health, whereas relatively few integrate these processes within the same plant–soil system. Moreover, differences among studies are often associated with variations in soil properties, climatic conditions, crop genotype, microbial strain compatibility, and management practices, making it difficult to generalize their agronomic performance across maize–cereal production systems. Consequently, the mechanisms responsible for improved crop performance are frequently described independently rather than as components of an integrated plant–soil regulatory network.

Another important limitation of the current literature is the relatively limited discussion of practical challenges affecting field implementation. Factors such as variability in microbial establishment, competition with native soil microbiota, formulation quality, persistence under field conditions, and site-specific responses remain insufficiently synthesized despite their importance for the successful adoption of microbial technologies in sustainable agriculture. Therefore, this review critically examines how BOF and microbial biostimulants influence crop yield, soil health, nutrient dynamics, and resilience to abiotic and biotic stresses in maize–cereal cropping systems, with particular emphasis on maize–wheat diversification. Rather than merely summarizing individual mechanisms, this review synthesizes current knowledge on the interactions among nutrient mobilization, rhizosphere processes, microbial activity, plant physiological regulation, and soil biological functioning. We further identify major knowledge gaps and practical challenges that should be addressed to improve the consistency, field applicability, and long-term sustainability of microbial BOF in cereal production systems.

## 2. Bio-Organic Fertilizers and Microbial Biostimulants in Maize–Cereal Systems: Concept and Functional Scope

### 2.1. Defining BOF and Microbial Biostimulants

BOF are biologically active inputs that support crop production through microbial and biochemical processes rather than solely through direct nutrient supply. They generally comprise PGPR, AMF, other beneficial microbes and biologically derived amendments that influence rhizosphere functioning and nutrient cycling. Unlike conventional mineral fertilizers, BOF primarily influence nutrient transformation, microbial activity, root functioning, and plant physiological responses rather than simply supplying nutrients [3,24,25]. This distinction becomes particularly evident in maize-cereal cropping systems. These systems are characterized by high nutrient demand, intensive agricultural management, and increasing exposure to environmental stresses. Collectively, these factors can progressively reduce soil biological functioning. Under these conditions, BOF function not only as nutrient suppliers but also as regulators of plant–soil–microbial interactions, thereby improving nutrient-use efficiency, stress tolerance, and long-term soil sustainability [5,9,20,26]. In maize-wheat and similar cereal-based rotations, this relevance extends beyond a single crop season because nutrient turnover, residue decomposition, and rhizosphere functioning can shape nutrient availability, microbial continuity, and early crop establishment in the following phase of the sequence [4,5,14,26]. The terminology surrounding BOF and biostimulants remains partially overlapping in the literature. Traditionally, BOF are defined as formulations containing living microorganisms that enhance nutrient availability and nutrient-use efficiency through biological processes such as nitrogen fixation, phosphorus solubilization, or nutrient mobilization. In contrast, biostimulants are products that improve plant growth, stress tolerance, nutrient uptake efficiency, or crop quality through physiological and biochemical regulation rather than direct nutrient supply alone [18,27,28]. Under current regulatory frameworks, particularly the European Union Fertilizing Products Regulation (EU 2019/1009), microbial plant biostimulants constitute a distinct category that includes microorganisms such as *Azospirillum* spp., *Azotobacter* spp., *Rhizobium* spp., mycorrhizal fungi, and *Trichoderma* spp. Consequently, many PGPR and AMF discussed in this review may function simultaneously as BOF, microbial biostimulants, and biocontrol-related agents depending on their dominant mechanism of action and application context [3,19]. Commercial microbial products currently used in cereal production systems include *Azospirillum*- and *Azotobacter*-based inoculants for nitrogen mobilization, *Bacillus*- and *Pseudomonas*-based formulations for nutrient solubilization and rhizosphere regulation, *Trichoderma*-based products for stress tolerance and biocontrol support, and AMF inocula containing species such as *Rhizophagus irregularis* and *Funneliformis mosseae*. These products are increasingly integrated into sustainable nutrient-management programs in maize and wheat systems, particularly under stress-prone environments where improved nutrient-use efficiency and rhizosphere resilience are required [19,27,28]. Because many microbial products simultaneously perform nutritional, physiological, and protective functions, rigid classification into BOF and microbial biostimulants, or biocontrol agents is often difficult and largely depends on their dominant mechanism of action or intended agricultural application. Despite recent regulatory advances, the classification of BOF, microbial biostimulants, and biocontrol agents remains a subject of ongoing discussion. The main challenge arises because many beneficial microorganisms simultaneously perform multiple biological functions. For example, a single *Bacillus* or *Trichoderma* strain may enhance nutrient availability, stimulate plant growth through phytohormone production, improve tolerance to abiotic stress, and suppress soil-borne pathogens. Consequently, the same microbial product may satisfy the functional definitions of a biofertilizer, a microbial biostimulant, and a biocontrol agent depending on the primary outcome being evaluated.

This functional overlap complicates comparisons among studies, creates inconsistencies in product classification and regulatory frameworks, and makes it more difficult to compare experimental results across different cropping systems. Rather than representing discrete categories, these biological products should be viewed as components of an integrated plant–soil management strategy in which multiple mechanisms frequently operate simultaneously [18,19,20,27,28]. To facilitate comparison among these overlapping functional categories, Table 1 summarizes the principal characteristics of BOF and microbial biostimulants, microbial biocontrol agents, and multifunctional microbial products, including their primary objectives, mechanisms of action, representative microorganisms, and major agronomic outcomes.

### 2.2. Functional Groups Relevant to Maize-Cereal Systems

A practical approach to classifying BOF is to categorize them according to the primary functions of their microbial components in nutrient transformation and plant growth promotion. One of the most widely recognized functional groups comprises nitrogen-fixing microorganisms that enhance nitrogen availability through biological nitrogen fixation and rhizosphere colonization. While symbiotic nitrogen fixation is typically associated with legumes, free-living and associative nitrogen-fixing bacteria, such as *Azotobacter* and *Azospirillum*, play a significant role in cereal production. These bacteria promote plant growth and can influence nitrogen availability in environments where nutrients are limited [3,29,30,31]. Phosphate-solubilising microorganisms represent another crucial functional group. This group is particularly important in maize-cereal systems. Here, phosphorus availability is often limited by soil chemical fixation. These microorganisms mobilize phosphorus using organic acids and enzymes, optimizing its use and enhancing plant uptake [27,32]. Their role is especially important in maize-wheat rotations, where high phosphorus demand exists across different crops. This can cause an imbalance between fertilizer application and availability for the plant. Similarly, potassium-mobilizing microorganisms can enable potassium solubilization, improving plant nutrition in soils with limited potassium availability [30,31]. Beyond macronutrient acquisition, BOF also includes microorganisms that are involved in the acquisition of micronutrients and the regulation of the rhizosphere. For example, siderophore producing microorganisms increase iron availability via chelation mechanisms and simultaneously restrict access to iron for competing pathogens, thus coupling nutrient acquisition to biocontrol functions [6,27,33]. Zinc and sulfur-solubilizing microorganisms further contribute to plant metabolism by increasing the availability of essential micronutrients required for enzymatic activity and physiological processes [34,35].

PGPR constitute one of the most multifunctional groups of beneficial microorganisms, influencing plant growth through multiple pathways, including nitrogen fixation, phosphate solubilization, siderophore production, phytohormone synthesis, and ACC deaminase activity. These traits enable PGPR to promote plant growth in both suitable conditions and under stress, particularly in cereal crops like maize and wheat [19,36,37,38]. In addition, combining PGPR with non-microbial biostimulants may further enhance their beneficial effects through the activation of cytokinin-dependent signaling pathways [39,40]. Another integral component of BOF and microbial biostimulants is AMF, which establish symbiotic associations with plant roots and increase the effective absorptive surface area through extensive hyphal networks. This enhances the uptake of phosphorus, micronutrients, and water [3,17,41]. In addition to their nutritional benefits, AMF contribute to soil aggregation and structural stability through glomalin-related processes and hyphal-mediated soil binding [42,43]. These functions play an important role in improving plant resilience to abiotic stresses such as drought and salinity, while also contributing to tolerance against biotic stress conditions through enhanced rhizosphere functioning and plant defense responses [44,45]. Collectively, these findings emphasize the importance of their role in nutrient uptake, stress tolerance and enhanced soil structure, a conclusion that is in line with the broader research results in the field of cereal systems. In rotation-based systems, these functions may support not only the current maize or wheat crop but also the continuity of rhizosphere microbial activity across crop transitions [42,46,47].

### 2.3. From Nutrient Supply to Stress Modulation

One of the defining characteristics of BOF is their ability to regulate multiple biological functions simultaneously. Unlike conventional fertilizers, which are mainly used to address nutrient deficiencies, bio-organic inputs can simultaneously influence nutrient acquisition, plant physiology, and stress tolerance. PGPR enhance plant performance by stress-induced ethylene accumulation, promoting root development through phytohormone production, and increasing antioxidant activity under adverse environmental conditions [27,44,48]. These mechanisms are clearly relevant to maize-cereal systems exposed to drought, salinity, and nutrient stress. Similarly, microbial traits that improve nutrient availability, such as phosphate solubilization and siderophore production, also help to promote plant health and resilience in challenging conditions. AMF and other beneficial microorganisms further enhance stress tolerance by improving water uptake, maintaining ionic balance, and stabilizing plant metabolic processes under unfavorable environmental conditions [28,44,49]. It is also important to recognize the considerable overlap among these biological functions. A single microbial group may simultaneously function as a biofertilizer, microbial biostimulant, and biocontrol agent. For example, PGPR improve nutrient acquisition while simultaneously promoting induced systemic resistance and suppressing pathogen development, illustrating the complex interactions between plants and beneficial microorganisms [50,51]. This multifunctionality is particularly relevant in maize-cereal systems, where biotic and abiotic stresses frequently co-occur and interact. Taken together, these observations illustrate the biological basis for the multifunctionality of BOF, providing the mechanistic foundation for the integrated plant–soil interactions discussed in the following sections. This perspective helps explain their role in sustainable agriculture, particularly under conditions where crop productivity depends not only on nutrient availability but also on resilience to environmental stress and maintenance of soil health.

### 2.4. Specific Relevance of BOF in Maize–Wheat Rotation Systems

The beneficial effects of BOF described throughout this review are broadly applicable to many cropping systems; however, their agronomic significance and practical implementation are particularly evident in maize–wheat rotations because of the unique ecological continuity created by successive cereal cultivation. Compared with continuous cereal systems, maize–wheat rotations provide greater crop diversity, different rooting patterns, and more heterogeneous residue inputs, thereby creating a more dynamic rhizosphere environment that supports microbial activity and nutrient cycling [13,14,15,16]. Consequently, microbial inoculants introduced during one crop may influence not only the current growing season but also soil biological functioning, nutrient availability, and rhizosphere processes affecting the subsequent crop. In contrast, long-term rice–wheat systems are frequently associated with soil degradation, nutrient imbalances, residue management challenges, declining soil biological activity, and increased environmental pressure resulting from repeated flooding–drainage cycles and intensive fertilizer use [10,11]. Replacing rice with maize in diversified maize–wheat rotations has therefore emerged as a promising strategy for improving resource-use efficiency while reducing water consumption, enhancing residue decomposition, and promoting more favorable soil aeration and microbial activity [12,13,14,15,16].

Under these conditions, BOF may provide additional benefits by enhancing nutrient mobilization, stimulating rhizosphere microbial communities, improving soil aggregation, and increasing nutrient-use efficiency across successive crop cycles. Their multifunctional nature is particularly valuable because nutrient mobilization, stress buffering, and microbial regulation occurring during the maize phase may contribute to improved soil functionality and early crop establishment during the subsequent wheat phase. Likewise, residue decomposition following maize harvest provides additional organic substrates that can support beneficial microbial communities and strengthen nutrient cycling before wheat establishment. Nevertheless, relatively few studies have directly compared the long-term performance of BOF in maize–wheat rotations with continuous rice–wheat systems under comparable environmental conditions. Future research should therefore evaluate how crop diversification influences microbial persistence, nutrient dynamics, soil biological functioning, and the cumulative benefits of BOF across multiple rotation cycles. Such studies will improve our understanding of the system-specific advantages of microbial technologies and support the development of sustainable nutrient-management strategies for diversified cereal production. Taken together, the concepts and functional characteristics discussed in this section provide the conceptual framework for understanding the biological mechanisms through which BOF influence crop performance. The following section therefore examines these mechanisms in greater detail, emphasizing how they collectively regulate plant growth, rhizosphere functioning, and soil biological processes.

## 3. Mechanisms Through Which Bio-Organic Fertilizers Influence Crop Performance

The multifunctional properties of BOF introduced in Section 2 are expressed through a network of interconnected biological mechanisms operating at the rhizosphere, plant, and soil-system levels. Rather than acting through a single pathway, microbial inoculants and biologically active amendments simultaneously regulate nutrient transformation, root development, rhizosphere interactions, physiological balance, and stress responses, ultimately determining crop performance [21,33,47]. In maize–cereal systems, where productivity is frequently constrained by high nutrient demand, repeated cultivation, and increasing environmental stresses, these mechanisms collectively influence nutrient-use efficiency, plant resilience, soil biological functioning, and yield stability [3,8,16,49]. The following sections examine these mechanisms individually while emphasizing their integrated contribution to sustainable crop production. BOF, including PGPR, AMF, nutrient-mobilizing microorganisms, and biologically derived amendments, regulate crop performance through multiple interconnected pathways. These include nutrient mobilization, root architecture modification, rhizosphere regulation, phytohormonal modulation, antioxidant and osmotic regulation, and biocontrol-related defense priming. Together, these mechanisms improve nutrient uptake, water balance, microbial functioning, and plant defense readiness, ultimately resulting in higher nutrient-use efficiency, improved crop vigor, enhanced stress tolerance, better soil health, and more stable yield performance.

**Figure 1:**
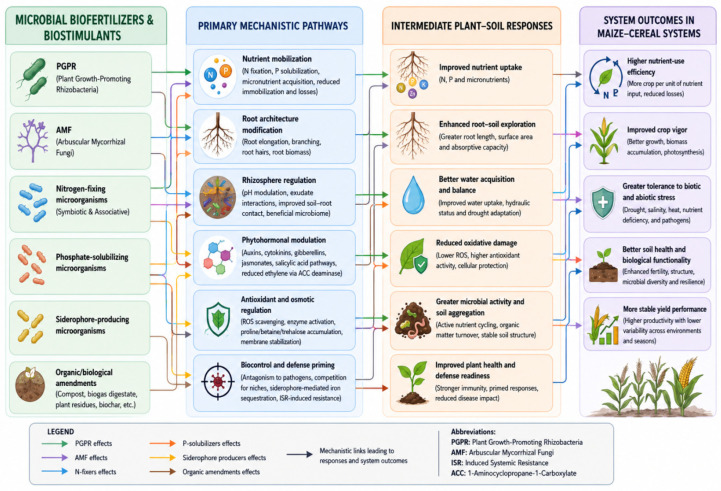
Conceptual framework illustrating the mechanisms by which BOF and microbial biostimulants enhance crop performance in maize-cereal systems.

Figure 1 summarizes these responses as an integrated system rather than as isolated processes. In practice, nutrient acquisition, physiological buffering, and soil biological regulation tend to operate together, and their combined effects are what ultimately shape nutrient acquisition, stress buffering, and final crop performance. These mechanisms form the basis for the stress-related responses discussed in the following section.

### 3.1. Nutrient Mobilization and Nutrient Use Efficiency

One of the primary mechanisms through which BOF improve crop performance is the enhancement of nutrient acquisition and nutrient-use efficiency. Beneficial microorganisms contribute to this process through biological nitrogen fixation, phosphorus solubilization, siderophore-mediated micronutrient acquisition, rhizosphere acidification, and stimulation of root–soil contact processes [33,52,53]. These functions are particularly important in maize–cereal production because maize, wheat, and related crops have high nitrogen and phosphorus requirements, whereas substantial proportions of applied nutrients remain unavailable because of immobilization, leaching, or volatilization [3,15,41]. Phosphate-solubilizing microorganisms improve phosphorus availability through the secretion of organic acids and phosphatases, which mobilize sparingly soluble inorganic phosphates and mineralize organic phosphorus fractions. This mechanism is particularly important in intensively managed soils, where phosphorus is often present but chemically inaccessible to roots [32,41,54]. Similarly, nitrogen-fixing and associative rhizobacteria may increase nitrogen availability while simultaneously improving root development and rhizosphere activity, thereby enhancing the efficiency with which cereals exploit soil nutrient pools [4,30,52]. Siderophore-producing microorganisms further support plant nutrition by enhancing iron mobilization and uptake and, in some cases improving zinc and other micronutrient acquisition, which contributes not only to plant metabolism but also to greater resilience under stress [6,27,55]. AMF further enhance nutrient-use efficiency by extending the effective absorptive surface area of roots through extensive extraradical hyphal networks. These hyphae enable plants to exploit soil volumes beyond the nutrient-depletion zone surrounding the root system, thereby improving phosphorus acquisition and the uptake of other relatively immobile nutrients, particularly in phosphorus-deficient soils [3,41].

These biological processes do not operate independently but interact with root architecture, rhizosphere chemistry, and microbial community dynamics to influence nutrient acquisition. Nevertheless, improvements in nutrient-use efficiency reported across studies vary considerably, indicating that microbial performance depends strongly on soil nutrient status, crop genotype, climatic conditions, and compatibility between introduced microorganisms and native soil microbiota. Consequently, nutrient mobilization should not be viewed as a universally predictable outcome of BOF application but rather as a context-dependent process whose effectiveness is determined by the characteristics of the entire plant–soil system [3,27,41,52,53].

### 3.2. Root Development and Rhizosphere Modification

Another critical pathway involves modifications of root architecture and rhizosphere structure. Root system architecture strongly influences nutrient and water acquisition, and many biological amendments enhance crop performance by modifying belowground development rather than merely supplying nutrients. One important aspect of PGPR is their ability to influence root features, i.e., elongation, branching, and root hair development. This is achieved by PGPR producing phytohormones such as indole-3-acetic acid (IAA) and related compounds that function as signals in the plant’s hormonal network [27,29,55,56]. These changes enhance the plant’s ability to explore soil resources more effectively, which is particularly advantageous in maize-cereal systems under nutrient limitation or intermittent drought [2,56,57,58]. Microbial metabolites may also alter the rhizosphere by affecting pH, mobilizing nutrients and affecting root exudate and microbial community composition. Such changes could generate more favorable micro-environments for nutrient turnover and beneficial root–microorganism interactions [3,28,53]. AMF contribute to this process by physically linking roots to a larger volume of soil, and some microbial consortia may promote co-colonization or synergistic establishment, thus increasing the stability of plant-microorganism associations under field conditions [28,42,44].

Although improvements in root development are consistently reported, their magnitude differs substantially among studies. Such variability probably reflects differences in soil physical properties, water availability, microbial colonization efficiency, and host genotype. These observations suggest that rhizosphere modification represents a dynamic biological process rather than a fixed response to microbial inoculation. Future studies should therefore focus on identifying the environmental and biological factors that determine successful root colonization and long-term rhizosphere stability under field conditions [28,42,56,59].

### 3.3. Hormonal and Biochemical Regulation

Beyond nutrient dynamics and root development, BOF modulate crop performance through hormonal and biochemical regulation. Beneficial rhizobacteria and microbial biostimulants can alter endogenous phytohormonal balance, including auxins, cytokinins, gibberellins, jasmonates, and salicylic acid-related pathways, thereby influencing growth regulation, defense readiness, and plant adaptation to stress [27,28,48]. One highly pertinent mechanism involves ACC deaminase activity, through which certain PGPR reduce stress-induced ethylene accumulation. Because excess ethylene under stress can inhibit root growth and reduce plant performance, microbial reduction in this signal represents an important route to improved tolerance and continued growth under adverse conditions [37,44]. Beneficial microorganisms may boost antioxidant defense systems and osmotic adjustment at the biochemical level. Reviews on the impact of stress suggest that microorganisms linked to plants can boost the production of metabolites, (e.g., proline, betaine and trehalose) and increase the activity of antioxidant enzymes (e.g., superoxide dismutase and catalase) [44,60]. These responses help stabilize membranes, reduce oxidative damage, and sustain physiological function during stress. In cereal crops, where yield losses often result from disruptions in photosynthesis, water relations, and metabolic balance, these biochemical effects can help maintain photosynthetic activity, membrane stability and plant growth under stress rather than simply promoting biomass accumulation under favorable conditions [6,43,61]. Despite substantial evidence supporting microbial regulation of phytohormones and antioxidant metabolism, the relative contribution of individual signaling pathways remains poorly understood. Hormonal responses are highly interconnected and frequently influenced by environmental conditions, making it difficult to attribute improved stress tolerance to a single mechanism. This complexity suggests that BOF-induced physiological regulation should be interpreted as the outcome of coordinated signaling networks rather than isolated hormonal responses [27,44,48,60]. Another important mechanism contributing to the effectiveness of BOF is the activation of ISR. Unlike direct antimicrobial activity, ISR enhances the capacity of plants to respond more rapidly and effectively to subsequent pathogen or herbivore attack through physiological priming rather than constitutive defense activation. PGPR and beneficial fungi, particularly *Trichoderma* spp., stimulate jasmonic acid- and ethylene-dependent signaling pathways, leading to enhanced expression of defense-related genes, increased production of defensive metabolites, and faster activation of plant immune responses following stress perception. This primed physiological state allows plants to maintain normal growth under favorable conditions while responding more efficiently when challenged by pathogens or insect herbivores. Consequently, ISR represents an important mechanistic link between rhizosphere colonization and improved resilience against multiple biotic stresses in maize–cereal systems [20,27,50,51].

### 3.4. Microbial Mediation of Soil Biological Functions

BOF modifies soil biological functions that are critical to long-term productivity and thus affect crop performance at the system level. Soil microbial biomass, enzyme activity, organic matter turnover, and aggregate stability all help determine how well soils retain nutrients, support root growth, and buffer crops against variable field conditions [16,20,62]. Beneficial microorganisms can stimulate nutrient cycling, alter the community composition of rhizosphere, and contribute to more functionally active soil ecosystems, particularly when combined with organic amendments or biologically active materials [3,63,64]. These changes are accompanied by shifts in the functional composition of rhizosphere microbial communities that enhance organic matter mineralization, extracellular enzyme activities, and synchronization between nutrient release and plant demand, thereby improving the efficiency of nutrient cycling within the soil microbiome. AMF are particularly important under these conditions because their hyphal networks contribute to soil aggregation and structural stability, partly through glomalin-related processes that help stabilize soil particles and improve the physical environment for roots and microorganisms [17,42,43,44]. Microbial inoculants may also interact with organic substrates to enhance decomposition and mineralization and to retain nutrients in biologically active pools rather than in forms that are rapidly lost. Although improvements in soil biological activity are frequently reported, their persistence under long-term field conditions remains insufficiently documented. Most available studies are based on relatively short experimental periods, making it difficult to determine whether observed changes in microbial communities and soil functionality remain stable across successive cropping cycles. Long-term field validation is therefore required before the broader agronomic benefits of BOF on soil health can be fully established [16,46,63,64,65].

### 3.5. Mechanistic Integration: From Plant Response to System Performance

The mechanisms described above are not independent of one another. Crop responses are usually the result of the interaction of these processes rather than any single mechanism alone. Improved root architecture enhances rhizosphere exploration, nutrient acquisition, and interactions with beneficial microorganisms, while microbial-mediated nutrient mobilization further supports plant growth and physiological performance. At the same time, hormonal regulation and enhanced soil biological activity contribute to greater stress buffering and long-term stabilization of plant–soil interactions [8,27,63]. This mechanistic integration helps to explain the often context-dependent but potentially large effects of BOF on crop productivity especially under stressful conditions [37,41,43,60,61]. In maize-cereal systems this means that yield responses should not be taken as the simple consequence of increasing the amount of nutrients. Instead, they reflect a coordinated change in plant–soil function where nutrient-use efficiency, physiological buffering and microbial support jointly influence crop performance [3,49,65,66]. Overall, the evidence indicates that the agronomic value of BOF emerges from the interaction of multiple biological processes rather than from any single mechanism. This systems-based perspective also explains why inconsistent field responses are commonly observed despite positive results under controlled conditions. Future research should therefore prioritize integrated studies combining plant physiology, rhizosphere ecology, soil microbiome analysis, and long-term field experimentation to better explain context-dependent responses and improve the predictability of microbial technologies in cereal production systems [49,65,66,67,68]. In maize-wheat and related rotational systems, this mechanistic integration is of clear relevance in because improvements in nutrient mobilization, rhizosphere activity, and soil biological regulation may influence both the current crop and the nutrient status, microbial activity, and structural condition of the soil for the following crop in the sequence [5,15,16]. These integrated mechanisms may also extend to aboveground interactions, where changes in plant physiology and chemistry can influence herbivore responses. Together, these interconnected mechanisms explain how BOF influence plant performance under both favorable and stressful environmental conditions. Building upon this mechanistic framework, the following section examines how these processes contribute to improved tolerance against abiotic and biotic stresses in maize–cereal production systems.

## 4. Bio-Organic Fertilizers Under Abiotic and Biotic Stress: Current Knowledge, Mechanisms, and Research Gaps in Maize–Cereal Cropping Systems

The biological mechanisms described in Section 3 become particularly important under stressful conditions, where the coordinated regulation of nutrient acquisition, root development, rhizosphere interactions, and plant physiological responses largely determines crop resilience. In maize–cereal production systems, crops are increasingly exposed to multiple interacting stresses, including drought, salinity, heat, nutrient imbalance, soil-borne pathogens, foliar diseases, and herbivorous insects. These stresses frequently occur simultaneously and disrupt nutrient uptake, water balance, plant metabolism, and ultimately yield stability. Under these conditions, the multifunctional nature of BOF enables them to mitigate stress not through a single mechanism but through the coordinated action of microbial-mediated nutrient mobilization, physiological regulation, antioxidant defense, and rhizosphere interactions [2,4,5,13,26,65]. Under abiotic stress, beneficial microorganisms and related bio-organic inputs enhance plant tolerance by improving root growth, nutrient acquisition, osmotic adjustment, and antioxidant defense. PGPR can reduce stress-induced ethylene accumulation through ACC deaminase activity, while AMF improve water uptake, ion balance, and nutrient transport under drought and salinity conditions [27,43,44,69]. Together, these responses help stabilize plant metabolism and reduce oxidative damage. This situation is especially serious in maize, which is highly sensitive to water and temperature stress [6,69,70].

Under biotic stress, BOF contribute to plant protection through pathogen suppression, siderophore-mediated competition, antimicrobial metabolite production, and ISR. PGPR, PGPF and AMF may reduce pathogen establishment by competing for nutrients and niches in the rhizosphere, enhancing nutrient-mediated plant defense, and priming plant immune pathways that improve resistance to infection and related growth challenges. As a result, the same microbial traits that support nutrient uptake may also contribute to plant health, ISR, and biologically based disease management, thereby enhancing crop resilience and supporting sustainable crop production [3,20,51,71,72]. Herbivorous insects should also be included within this biotic stress framework. Beyond their role in pathogen suppression, PGPR, AMF, and *Trichoderma* spp. can indirectly influence insect herbivores by altering plant defense status, nutrient profiles, and host suitability. Such plant-mediated effects may reduce feeding damage, oviposition, or larval performance. However, outcomes depend on the crop, microbial strain and herbivore species involved. This dimension is particularly relevant in maize-based systems, where beneficial soil microorganisms have been shown to modify aboveground herbivore interactions as part of a broader plant–soil defense response [9,26]. These responses should not be viewed separately. Nutrient mobilization, hormonal modulation, antioxidant regulation and defense priming often work together, giving BOF the capacity to buffer multiple forms of stress at the same time. This multifunctionality is particularly relevant in maize-cereal systems where the stress tolerance, soil functioning and stability of yield is based on integrative plant–soil response and not on single traits alone [44,49,63]. As a result, the role of BOF under stress may be described as the coordinated modulation of both abiotic and biotic stress responses within a shared plant–soil–microorganism framework. This becomes more critical in maize-wheat diversification systems, where the benefits of stress buffering may extend beyond immediate crop protection by helping preserve nutrient balance, microbial activity, soil structure, and the productive continuity of the rotation [16,31,47,65]. Although substantial progress has been made in understanding the role of BOF under stressful conditions, current knowledge remains uneven across different stress categories. Most available studies have focused on drought and salinity because these represent the most widespread constraints limiting cereal production worldwide. Comparatively fewer investigations have examined responses to heat stress, nutrient imbalance, flooding, or combinations of multiple abiotic stresses.

Similarly, while considerable attention has been devoted to soil-borne pathogens, much less information is available regarding the influence of microbial BOF on aboveground diseases, insect herbivores, and simultaneous biotic and abiotic stress scenarios. Since crops growing under field conditions are commonly exposed to multiple interacting stresses rather than individual stress factors, greater emphasis should be placed on integrated experimental approaches that evaluate microbial performance under realistic environmental conditions. Such studies will be essential for improving our understanding of the consistency, resilience, and practical applicability of BOF in maize–cereal production systems [8,19,20,21,44,49].

It is clear that BOF strongly impacts the performance of the plant through a number of interconnected pathways. Moreover, this process is characterized by its dualistic nature consisting of abiotic and biotic stress factors. In the context of abiotic stress, they have been observed to enhance osmotic adjustment, antioxidant defense, ion homeostasis, and nutrient uptake. Together, these observations indicate that the role of BOF under stressful conditions should be interpreted as part of an integrated plant–soil regulatory network rather than as isolated physiological effects. In maize–cereal systems, their contribution is particularly relevant under combined stress scenarios, where coordinated rhizosphere activity, nutrient regulation, and stress buffering may help stabilize crop performance and soil functionality across growing cycles.

**Figure 2:**
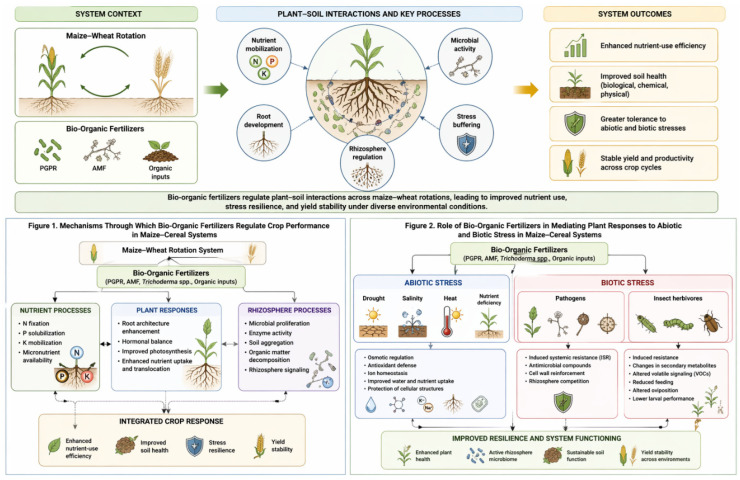
Illustration of the coordinated mechanisms through which BOF improve tolerance to abiotic and biotic stresses in maize–cereal production systems. Arrows indicate the temporal sequence.

Although the mechanisms described above are presented separately for clarity, they rarely operate independently under field conditions. In maize–wheat rotation systems, microbial-mediated nutrient mobilization, root architectural modification, phytohormone regulation, antioxidant activation, induced systemic resistance, and rhizosphere microbial interactions function as interconnected processes that collectively determine plant performance. Improved nutrient availability promotes root development, which in turn promotes more effective microbial colonization and improves water and nutrient acquisition. Simultaneously, microbial regulation of plant hormonal balance and antioxidant metabolism strengthens tolerance to abiotic stresses while priming defense responses against soil-borne and foliar pathogens. These complementary interactions generate synergistic rather than merely additive effects, contributing to improved crop establishment, greater resilience to multiple environmental constraints, and enhanced productivity across successive maize–wheat cropping cycles. Consequently, evaluating individual mechanisms in isolation may underestimate the overall contribution of BOF under practical field conditions, where plant, microbial, and soil processes interact continuously throughout crop development [3,16,20,27,49,63,64,65]. A visual summary is shown in Figure 2.

The major microbial groups discussed in this review, together with their representative microorganisms, primary biological functions, target abiotic and biotic stresses, and reported effects in maize and wheat production systems, are summarized in Table 2.

## 5. Impacts on Soil Health, Nutrient Dynamics, and Yield Stability

Beyond their direct effects on individual plants, the biological mechanisms described in the preceding sections collectively influence soil functioning, nutrient dynamics, and the long-term sustainability of maize–cereal production systems. The improvements in nutrient mobilization, rhizosphere activity, microbial interactions, and stress resilience described above also extend to the soil environment, where they influence microbial biomass, enzyme activity, soil aggregation, organic matter turnover, and nutrient cycling. These processes are closely associated with improved soil fertility, root-zone functioning, and the long-term stability of crop production [49,52,53,61,63,67]. At the soil level, beneficial microorganisms and biologically active amendments further enhance microbial activity, improve soil aggregation, and promote more efficient nutrient transformation processes, thereby strengthening the resilience of the entire plant–soil system. However, the extent of these improvements varies considerably among soil types, reflecting differences in soil texture, organic matter content, pH, and the composition of resident microbial communities, all of which influence microbial establishment and activity [16,46,63,64]. AMF are particularly useful because the hyphal networks of AMF improve soil structure and help stabilize biologically active zones around roots, while other microbial groups are involved in decomposition, mineralization and rhizosphere-mediated nutrient turnover [42,69]. These processes enhance the soil not only physically but also in terms of microbial activity, nutrient turnover and root zone functioning. BOF also affects nutrient dynamics by improving the mobilization, uptake, and retention of important nutrients, particularly nitrogen and phosphorus. Through nitrogen fixation, phosphorus solubilization, and root-associated nutrient capture, they can improve nutrient-use efficiency and reduce the mismatch between nutrient availability and plant demand in intensive cereal systems [4,5,10,15,30,54]. This is highly relevant in maize-cereal production, where repeated fertilizer use is often associated with low recovery efficiency and progressive decline in soil quality. Although improvements in soil biological activity, nutrient cycling, and yield stability have been widely reported following BOF application, these responses are not universal. Their magnitude depends strongly on soil physicochemical properties, initial soil fertility, organic matter content, climatic conditions, crop genotype, and the composition of native microbial communities. For example, greater benefits are often observed in degraded or nutrient-deficient soils where microbial activity has been reduced, whereas responses may be smaller in fertile soils that already possess high biological functionality. Likewise, improvements in soil structure and microbial diversity frequently require repeated applications over multiple cropping seasons rather than a single growing cycle, indicating that many of the benefits associated with BOF accumulate gradually over time rather than appearing immediately.

**Figure 3:**
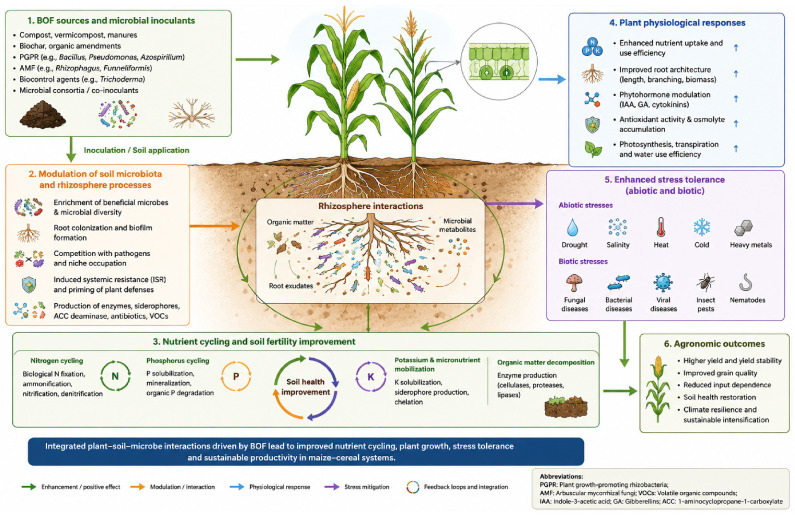
Integrated framework illustrating the interactions among BOF, soil microbiota, nutrient cycling, plant physiological responses, and resilience to abiotic and biotic stresses in maize–cereal cropping systems. BOF enhance rhizosphere microbial activity, stimulate nutrient cycling and soil fertility, regulate plant physiological processes, and activate stress-response mechanisms, ultimately improving crop productivity, soil health, and agricultural sustainability.

These observations suggest that the agronomic value of BOF should not be evaluated solely on the basis of short-term yield increases. Instead, their principal contribution may lie in enhancing the long-term resilience of plant–soil systems by maintaining soil biological activity, improving nutrient-use efficiency, and reducing yield fluctuations under variable environmental conditions. Consequently, future studies should emphasize long-term field experiments that evaluate the persistence of microbial communities, changes in soil functionality across successive crop rotations, and the consistency of yield responses under contrasting environmental conditions [3,4,5,16,42,49,63,64,65]. Evidence from long-term field experiments increasingly suggests that the benefits of BOF extend beyond immediate improvements in crop growth and nutrient uptake. Repeated applications across successive cropping seasons have been associated with gradual enhancement of soil biological activity, stabilization of microbial communities, improved nutrient cycling, and greater resilience of plant–soil systems under variable environmental conditions. Unlike short-term greenhouse studies, long-term field experiments are also better suited to evaluate the persistence of introduced microorganisms, their interactions with native soil microbiota, and the cumulative effects of BOF on soil quality and crop productivity across multiple crop rotations. Consequently, greater emphasis should be placed on long-term, multi-location field validation to determine the durability, consistency, and practical applicability of microbial technologies under commercial farming conditions [5,16,49,63,64,65]. The integrated relationships among BOF inputs, soil microbiota, nutrient cycling, plant physiological regulation, stress tolerance, and agronomic outcomes in maize–cereal systems are summarized in Figure 3.

## 6. Context Dependency, Optimization Challenges, and Knowledge Gaps

Although BOF and microbial biostimulants have demonstrated considerable potential for improving crop productivity and soil health, their performance remains highly context dependent and often varies among production systems. Responses are influenced by soil physicochemical properties, crop genotype, microbial strain compatibility, climatic conditions, and agricultural practices. This variability results from the combined influence of microbial strain compatibility, soil physicochemical properties, native microbial competition, climatic conditions, crop genotype, inoculum formulation, application timing, and management practices, all of which determine microbial establishment, persistence, and functional activity after field application. In many cases, differences observed between greenhouse and field performance are associated not with lack of efficacy, but with the complexity of rhizosphere interactions, microbial establishment, and competition with native soil microbiota under open-field conditions [64,70]. These factors highlight the importance of site-specific optimization and long-term field validation for improving the consistency and scalability of microbial-based agricultural inputs. Overall, published studies consistently demonstrate the potential of BOF to improve nutrient-use efficiency, soil health, and crop productivity; however, the magnitude of these responses differs considerably among experiments. Studies conducted in degraded, nutrient-deficient, or stress-prone environments generally report greater improvements than those performed in fertile soils or under favorable growing conditions. Likewise, microbial inoculants often exhibit stronger effects under controlled greenhouse conditions than under field environments, where interactions with native microbial communities and environmental variability become increasingly important. These apparently contrasting findings should therefore be interpreted as evidence of the context-dependent nature of microbial technologies rather than as contradictions regarding their biological effectiveness [3,4,5,16,19,27,49,64,70]. One major limitation is the variability in the quality and formulation of inoculants. Performance may be affected by shelf life, contamination risk, carrier material, and the persistence and functionality of introduced microorganisms within heterogeneous soils. This is especially true for microbial products designed to serve as both BOF and biocontrol agents because multifunctionality does not necessarily translate to reliability [73,74,75]. Another limitation is that much of the available evidence is still based on short-term experiments, single-stress scenarios or simplified greenhouse systems. However, maize-cereal cropping systems are constrained by a series of interacting limitations including nutrient limitations, abiotic stress, pathogens and the variability of soil biological conditions. Thus, many published responses remain difficult to generalize, particularly when studies do not sufficiently take into account plant genotype, resident soil microbiota, or longer-term responses in the field [49,75,76]. There are also remaining important knowledge gaps in the design of microbial consortia and in understanding strain compatibility. It is not possible to determine the efficacy of a mixture based on the performance of individual strains. So, the synergistic or antagonistic interactions within formulations are not well understood [70,74,77]. Beyond biological efficacy, successful implementation of BOF depends on several practical considerations that remain insufficiently addressed in the current literature. Commercial adoption requires not only effective microbial strains but also reliable formulation technologies capable of maintaining microbial viability during production, storage, transportation, and field application. Carrier materials, shelf life, storage conditions, and compatibility with commonly used fertilizers and pesticides may substantially influence product performance before microorganisms even reach the rhizosphere. In addition, production costs, product availability, and economic return remain important factors influencing farmer adoption, particularly in large-scale cereal production systems where profitability strongly determines management decisions.

Consequently, successful commercialization requires coordinated improvements in formulation technology, quality assurance, economic feasibility, and extension programs that facilitate farmer confidence and technology transfer [19,20,27,28,73,74,75]. Another major challenge concerns the lack of international standardization in the classification, evaluation, and regulation of microbial products. Although recent regulatory frameworks have improved the distinction between microbial biostimulants and BOF, considerable differences still exist among countries regarding product registration, efficacy testing, quality standards, and labeling requirements. These inconsistencies complicate comparisons among published studies and may slow the global commercialization of innovative microbial technologies. Furthermore, microbial persistence after field application remains poorly understood because introduced microorganisms frequently compete with well-established native microbial communities whose composition varies among soils and climatic regions. Consequently, microbial consortia that perform successfully under one environmental condition may exhibit reduced establishment or inconsistent performance elsewhere. Future formulation strategies should therefore focus on designing environmentally adapted microbial consortia that remain functionally stable under diverse climatic conditions and cropping systems rather than relying on universally applicable inoculants [18,19,20,27,28,73,74,75,76,77]. The current literature also reflects differing perspectives regarding the primary mechanisms responsible for the agronomic benefits of BOF. Some studies attribute improved crop performance primarily to enhanced nutrient availability and nutrient-use efficiency, whereas others emphasize microbial regulation of plant physiology, hormonal signaling, rhizosphere interactions, or induced systemic resistance as the dominant drivers of plant growth and stress tolerance. These contrasting interpretations are not necessarily contradictory but rather reflect differences in soil properties, cropping systems, environmental conditions, microbial composition, and experimental design. Consequently, BOF should be viewed as multifunctional biological systems whose effectiveness depends on the coordinated interaction of multiple mechanisms rather than on any single process acting independently. Future research should therefore move beyond evaluating isolated mechanisms toward integrated studies capable of quantifying the relative contributions and interactions of biological, physiological, and ecological processes under realistic field conditions [3,16,20,27,49,63,64,65].

Future research should therefore move beyond isolated traits and short-term yield responses toward integrated, multidisciplinary approaches combining plant physiology, rhizosphere ecology, soil microbiome analysis, microbial formulation science, and long-term field experimentation. Greater emphasis should also be placed on evaluating microbial persistence, interactions with native microbiota, climate-dependent performance, and the design of robust microbial consortia capable of maintaining efficacy across contrasting environments. Emerging technologies such as metagenomics, metatranscriptomics, metabolomics, and other multi-omics approaches will provide unprecedented opportunities to unravel the complex interactions governing plant–microbe–soil systems and to identify biomarkers associated with successful microbial establishment and crop performance. Furthermore, advances in microbiome engineering and the development of synthetic microbial consortia may facilitate the design of functionally optimized inoculants tailored to specific soils, crops, and environmental conditions. The integration of microbial technologies with precision agriculture tools, including soil sensing, digital monitoring, artificial intelligence, and data-driven nutrient management, could further improve the efficiency and site-specific application of BOF under commercial farming conditions. Such interdisciplinary approaches will be essential for developing climate-resilient cropping systems capable of maintaining productivity, resource-use efficiency, and soil health under increasingly variable environmental conditions.

## 7. Conclusions and Future Perspectives

The evidence synthesized throughout this review demonstrates that different categories of BOF contribute to maize–wheat production through complementary rather than identical mechanisms. Plant growth-promoting rhizobacteria (PGPR) primarily enhance nutrient acquisition, phytohormone regulation, and root development, whereas arbuscular mycorrhizal fungi (AMF) contribute predominantly to phosphorus uptake, water acquisition, and soil structural improvement through extensive hyphal networks. Plant growth-promoting fungi (PGPF), particularly Trichoderma spp., play key roles in induced systemic resistance, pathogen suppression, and root stimulation, while multifunctional microbial consortia integrate several of these mechanisms simultaneously. Although the relative contribution of each BOF group varies with soil properties, crop genotype, environmental conditions, and management practices, the available evidence consistently indicates that combining complementary microbial functions generally provides greater and more stable improvements in nutrient-use efficiency, stress resilience, soil biological activity, and crop productivity than reliance on individual microbial groups. These findings highlight that the successful application of BOF in maize–wheat production systems depends on matching microbial functions to specific agronomic objectives and local environmental conditions rather than assuming universal effectiveness across all production environments [3,16,20,27,49,63,64,65]. Although many of the biological mechanisms discussed in this review are common to a wide range of crop species, their agronomic significance and practical application are strongly influenced by cropping-system characteristics. In maize–wheat production systems, successive cereal cultivation, contrasting rooting patterns, residue dynamics, and high nutrient demand create a distinct ecological environment in which BOF contribute not only to individual crop performance but also to nutrient cycling, rhizosphere continuity, and soil biological functioning across successive rotation cycles. Therefore, the novelty of this review lies not in describing mechanisms unique to maize or wheat, but in integrating current knowledge within the specific ecological and management context of diversified maize–wheat production systems. Future progress in this field will depend less on the identification of additional microbial strains and more on understanding how beneficial microorganisms function within complex plant–soil ecosystems under realistic agricultural conditions. Greater emphasis should be placed on elucidating microbial interactions with native soil microbiota, improving formulation technologies that enhance microbial persistence and shelf life, and developing functionally compatible microbial consortia capable of maintaining stable performance across diverse soils and climatic conditions.

Advances in multi-omics approaches, including metagenomics, metatranscriptomics, metabolomics, and systems biology, will provide valuable opportunities to better understand the mechanisms underlying plant–microbe–soil interactions and to identify reliable biomarkers associated with successful microbial establishment and crop response. Equally important will be long-term, multi-location field experiments integrating biological, agronomic, economic, and environmental assessments to validate the practical performance of BOF under commercial farming conditions. Such integrated research will facilitate the development of scientifically robust, economically viable, and environmentally sustainable microbial technologies for future maize–cereal production systems.

## Figures and Tables

**Table 1:** Comparative overview of BOFand microbial biostimulants, microbial biocontrol agents, and multifunctional microbial products relevant to maize–cereal cropping systems.

Category	Primary Objective	Main Mechanism(s)	Representative Microorganisms	Principal Outcome
BOF	Improve nutrient availability and nutrient-use efficiency	Biological nitrogen fixation, phosphorus solubilization, potassium mobilization, siderophore production	*Azotobacter*, *Azospirillum*, *Rhizobium*, phosphate-solubilizing *Bacillus*, *Pseudomonas*	Enhanced nutrient uptake and plant nutrition
Microbial biostimulants	Improve plant growth and stress tolerance	Phytohormone production, ACC deaminase activity, osmotic adjustment, antioxidant activation, rhizosphere regulation	PGPR, AMF (*Rhizophagus irregularis*, *Funneliformis mosseae*), *Trichoderma* spp.	Enhanced growth, nutrient-use efficiency and abiotic stress tolerance
Microbial biocontrol agents	Suppress plant pathogens and pests	Competition for nutrients and niches, antimicrobial metabolite production, mycoparasitism, induced systemic resistance (ISR)	*Trichoderma* spp., *Bacillus subtilis*, *Pseudomonas fluorescens*, *Streptomyces* spp.	Reduced disease incidence and improved plant health
Multifunctional microbial products	Simultaneously improve nutrition, growth, stress tolerance and plant protection	Combination of nutrient mobilization, phytohormonal regulation, ISR, rhizosphere colonization and microbiome modulation	Many PGPR, AMF and *Trichoderma*-based commercial products	Integrated improvement of plant–soil functioning

**Table 2:** Major microbial groups associated with bio-organic fertilizers and their reported functions in maize–cereal cropping systems.

Microbial Group	Representative Microorganisms	Primary Biological Functions	Major Target Stresses	Reported Agronomic Effects in Maize and Wheat
PGPR	*Azospirillum*, *Azotobacter*, *Bacillus*, *Pseudomonas*	Nitrogen fixation, phosphate solubilization, phytohormone production, ACC deaminase activity	Drought, salinity, nutrient deficiency, pathogens	Improved nutrient-use efficiency, root growth, stress tolerance, yield
AMF	*Rhizophagus irregularis*, *Funneliformis mosseae*, *Claroideoglomus etunicatum*	Enhanced nutrient uptake, hyphal networking, water acquisition	Drought, salinity, phosphorus deficiency	Improved phosphorus uptake, water relations, biomass, stress resilience
PGPF	*Trichoderma harzianum*, *Trichoderma asperellum*	Root colonization, ISR induction, enzyme production, pathogen suppression	Soil-borne diseases, drought	Enhanced disease resistance, root development, plant vigor
Plant-beneficial bacteria	*Bacillus subtilis*, *Pseudomonas fluorescens*	Antibiotic production, siderophore secretion, ISR, nutrient mobilization	Fungal pathogens, nutrient limitation	Reduced disease incidence, improved nutrient acquisition
Microbial consortia	PGPR + AMF + *Trichoderma* combinations	Complementary nutrient cycling, rhizosphere regulation, physiological priming	Combined abiotic and biotic stresses	Greater stability of plant performance and soil biological activity than single inoculants

## Data Availability

No new data were created or analyzed in this study.
